# A study on mechanism of resistance distribution characteristics of oxide-based resistive memory

**DOI:** 10.1038/s41598-018-35838-x

**Published:** 2019-01-22

**Authors:** Ji-Hyun Hur, Deok-kee Kim

**Affiliations:** Electrical Engineering, Sejong University, Neungdong-ro 209, Seoul, 05006 Republic of Korea

## Abstract

Although oxide-based resistive switching memory (OxRAM) is one of the strong next-generation high capacity memory candidates, it has the critical disadvantage that deviations of resistance levels is too severe to be adopted as a high capacity memory device. More specifically, it is known that the larger on/off current ratios in multi-level operated OxRAMs, the greater deviation of resistance levels from the targeted values. However, despite the seriousness of the problem there has been no concrete theoretical study on the underlying mechanisms of the phenomenon. In this paper, we introduce a theoretical model that clearly explain the underlying mechanism of making such characteristics of programmed resistance levels in multi-level OxRAMs. From this model, we can understand why there is a proportional relationship between resistance level and its deviation, and why it has such a specific range of proportionality constant measured experimentally. And this understanding can certainly reveal the true limitations of OxRAMs’s performance.

## Introduction

In recent decades, oxide-based resistance switching nanodevice, often called OxRAM (oxide-based resistive random access memory) has been the subject of numerous semiconductor memory manufacturers and academies due to its potential advantages of simple two terminal structure instead of three-terminals in conventional memory devices that results in good scalability, good switching endurance, and fast switching speed. OxRAMs usually have bi-layer oxide structures, in which a thin, nearly stoichiometric oxide layer (resistance switching layer) with higher resistivity and a thick, metal-rich layer with lower resistivity (base layer) are sandwiched by two electrodes^1–11^. OxRAMs show switching endurance of up to 10^11^, which is much larger than NAND flash comparable to DRAM and also mark faster switching speed, usually in the range of 100 ns, or even below the ns regime^4^. High switching speed of OxRAMs, combined with relatively low-voltage operation compared to NAND flash also allows for low program/erase power consumption which is suitable for low-power application.

Despite the advantages, OxRAMs have several major drawbacks to become a mass-produced high-capacity memory to replace NAND flash. The most important issue is the poor cycle-to-cycle uniformity of resistance levels. That is, It have been reported that wide distribution of resistances appear for high resistance levels (off-states) while quite uniform distribution for low resistance level (on-state)^3,6^. This tendency becomes clearer at multi-resistance level operation, where a larger deviations of resistance levels appear in the larger on/off ratios.

This problem of deviation in programmed resistance levels of OxRAMs can be mitigated to some extent by applying more complicated switching pulse application scheme with loss of switching speed^6^, but if we do not understand the true principles of the issue, it is impossible to figure out the true limitation of OxRAMs performance as a high-capacity memory. Based on that, we can find out whether the problem is intrinsic or can be almost completely solved with some efforts such as optimizations in processes and programming scheme. By taking these into account, the need to understand the underlying principles that cause the problem cannot be overemphasized.

With this motivation, we investigate in this paper the characteristics of distribution of programmed resistance levels in OxRAM and find out the mechanisms that cause the aforementioned phenomenon. To achieve this goal, we develop a theoretical model that derived only from the basic knowledge of statistical physics combined with the OxRAM’s resistance switching model without any kind of empirical factors. We show this model has remarkable qualitative and quantitative agreements with the experimental measurements.

## Results

### Multi-level switching experiment with Ta_2_O_5−x_/TaO_2−x_ OxRAM

**Figure 1:**
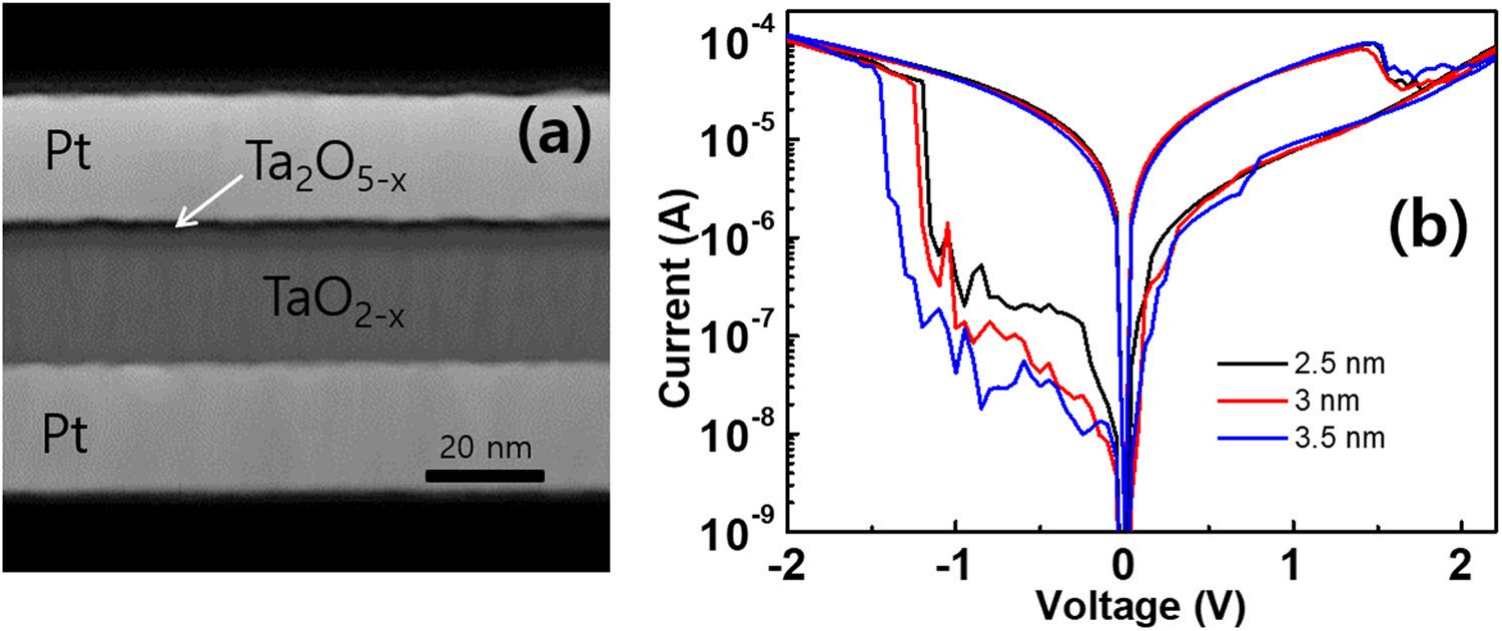
(**a**) Transmission electron microscopy image of fabricated Pt/Ta_2_O_5−x_/TaO_2−x_/Pt OxRAM with 50 ALD cycles for deposition of resistance switching layer of which resulting thickness is about 3 nm. (**b**) Experimental measurements of I–V curves for several atomic layer deposition number of cycles (40, 50, and 60) during deposition of resistance switching layer. The resulting switching layer thicknesses are about 2.5, 3, and 3.5 nm respectively. The switching voltages are applied with slow speed of sweep (5 s of period) from −2 V to 2.5 V.

We begin the study with an explanation of OxRAM fabrication procedure followed by the description of resistance distribution characteristics. The multi-resistance level switching experiments of OxRAM was conducted with the bi-layered tantalum oxide having Pt/ Ta_2_O_5−x_ /TaO_2−x_/Pt-structure^1,5,6^. The device was fabricated on a bottom electrode Pt layer followed by depositing the metal-rich, highly conductive TaO_2−x_ base layer of about 50 nm thickness using reactive sputtering of a Ta metal target in an oxygen and argon gas mixture. We then form the thin, insulating Ta_2_O_5−x_ layer by placing the pre-processed samples in a plasma oxidation chamber used for atomic layer deposition (ALD) of which number of cycles are varied to make different thicknesses of Ta_2_O_5−x_ layer. And finally, the Pt top electrode with the size of 500 × 500 nm^2^ is deposited on top of that. Normally, electrodes for OxRAM adopt noble metal such as Pt in order to minimize oxidation reaction between the electrode and oxide layer. The transmission electron microscopy (TEM) image of the fabricated OxRAM structure with 50 ALD cycles is shown in Fig. 1(a). Actually, switching of resistance state occurs in the resistance switching layer (Ta_2_O_5−x_), more specifically within a very narrow filament of which size is believed to be less than 100 nm diameter produced through a so called ‘forming’ process. It is known that the TaO_2−x_ base layer acts as a migration path and storage depot of oxygen ions and should have lower resistivity than Ta_2_O_5−x_ layer so that more portion of external voltage can be applied to Ta_2_O_5−x_ layer at off-state and actual resistance switching takes place there.

Figure 1(b) shows the difference in characteristics of the switching curves due to the changes in the number of ALD cycles and resulting changes in thickness of Ta_2_O_5−x_ layer. The measurements were made with a slowly varying sinusoidal voltage sweep of 5 s period. In our experiments, when ALD cycles were 40, 50, and 60, the thickness of the Ta_2_O_5−x_ layer was measured at 2.5 and 3, 3.5 nm, respectively. From the figure, we can see the asymmetry behavior at off-state caused by the Schottky barrier formed between the top electrode and the resistance switching layer, which is a typical feature for bi-layered OxRAMs^1^.

**Figure 2:**
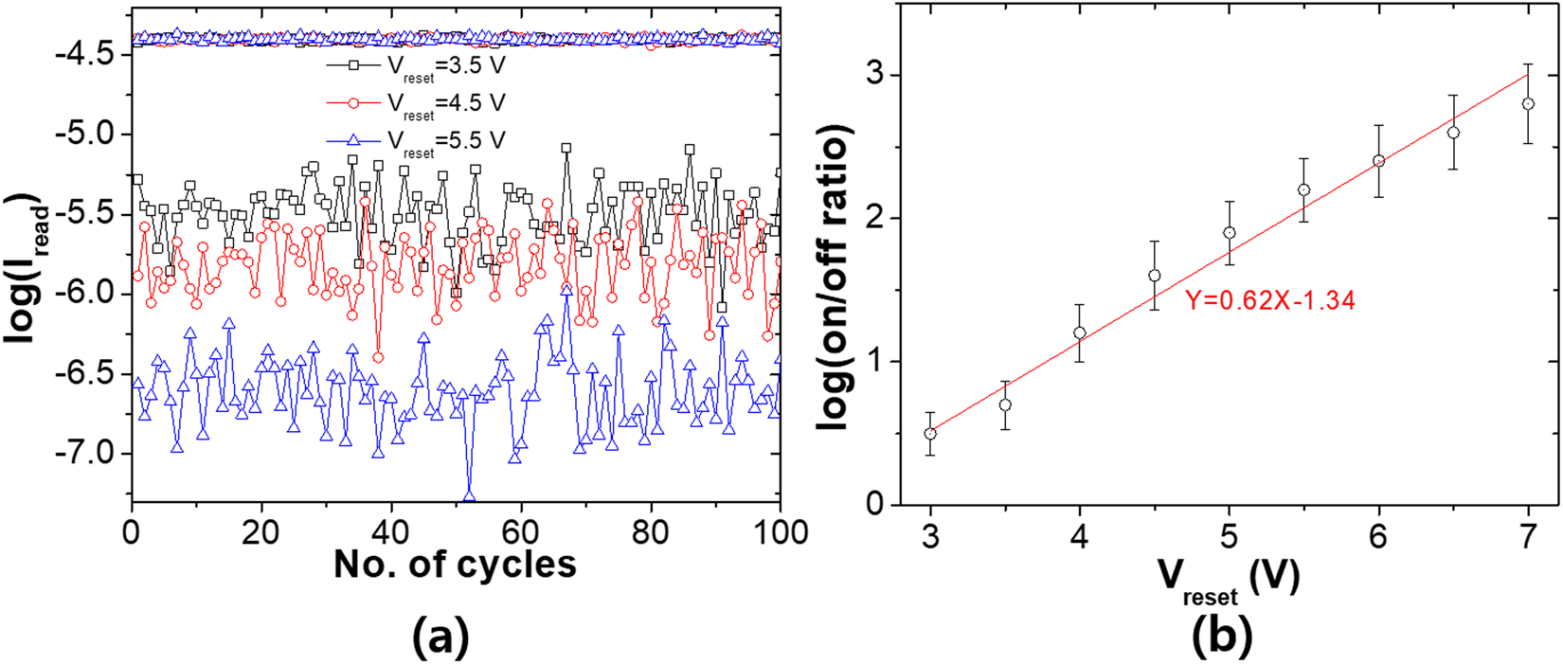
(**a**) Experimentally measured multi-level resistance switching results with the OxRAM of L = 3 nm. Different off-state resistances are created by applying different reset pulses (3.5, 4.5, 5.5 V) and repeated 100 cycles. The set/reset pulses are all 500 ns of duration time and the read is performed by applying 0.5 V pulse between each programming. (**b**) log-scale on/off ratio as a function of V_reset_ along with the linearly fitted line (red).

Switching of resistance state in mass-produced memories is performed by applying a positive (reset) pulse signals at the top electrode on the order of nanoseconds and multiple off-state programming is realized with a set of pulses with different voltage heights. The programming of multiple off-states with the fabricated (50 cycles of ALD) OxRAM are also obtained by varying height of reset pulse and the results are shown in Fig. 2(a). The reset pulse signals have 500 ns of duration time with an initial amplitude of 3.5 V and being increased by 1 V for every 250 μs to achieve stats having larger resistances. Meanwhile, the set pulse and read pulses are fixed at −5 V and 0.5 V respectively. Figure 2(b) shows the dependence of on/off current ratio (logscale) on reset voltages where the on/off ratio increases up to ~10^3^ as the reset pulse voltage increases. We can figure out that the logscale of on/off current ratio is almost linearly proportional to the reset pulse height of from 3 V to 7 V. One thing that we can also notice in Fig. 2(a) is that the deviations of off-state resistances are very large compared to that of on-state and increase as the resistances increase (I_read_ decreases).

**Figure 3:**
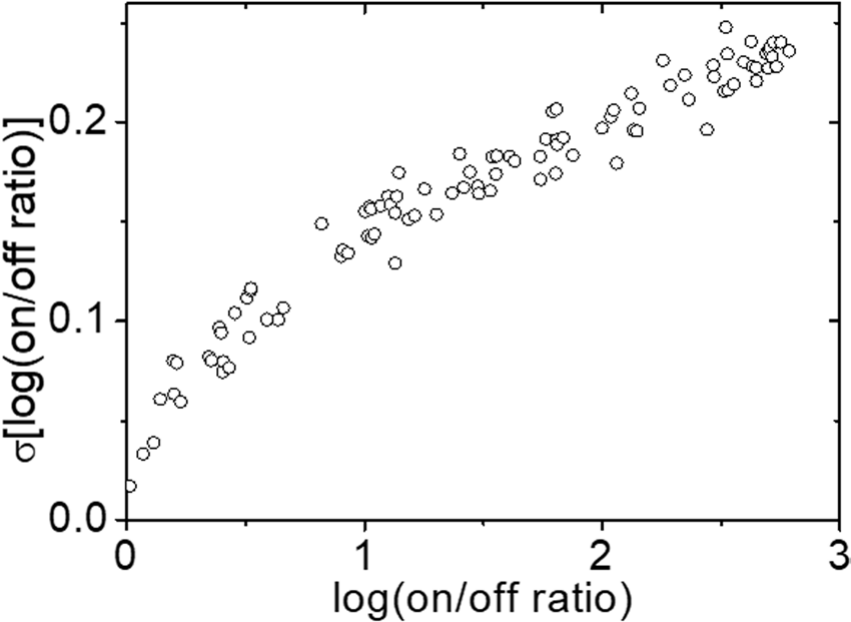
Plots for standard deviations of log-scale on/off current ratios as a function of log-scale of averaged on/off current ratios from the experimental measurements with the OxRAM of L = 3 nm.

In Fig. 3, we show how the standard deviation of the on/off current ratio in logscale (σ[log(on/off)]) depends on the on/off current ratio averaged for each resistance level measured with the same OxRAM device shown in Fig. 2. It can be clearly seen that σ[log(on/off)] increases proportionally from near 0 up to 0.25 when log(on/off) changes from 0 to 3. In order for OxRAMs to be more economically competitive, it is absolutely necessary to have higher memory capacities, which requires multi-level resistance operation that again demands large on/off current ratios. However, huge deviation of resistances from the targeted resistance is a major concern for implementation of multiple resistance-level operation because basically if there are more than a certain level of overlaps between neighboring resistance states, the states are read incorrectly resulting in memory reading error. Although there is a way to decrease deviation of resistances to some extent by applying modified programming schemes such as the multiple-pulse program scheme^6^, but it requires huge loss of programming speed and is far from the fundamental solution at all.

## Development of theory of resistance distributions

The starting point for explaining this kind of statistical phenomena with a theoretical model should be the basic OxRAM switching model that can explain a single cycle of resistance switching as given in ref.^1^ in detail. As described in Fig. 1(a), an OxRAM normally has a double oxide layer structure, sandwiched by top and bottom electrodes which can be modeled as a series-connected circuit of resistors (base layer and resistance switching layer) and a variable Schottky barrier. The Schottky barrier is formed between the filament and the top electrode of which barrier height is variable by a stoichiometric state of the filament, i.e., a fractional change of oxygen/metal ions in the filament^1,2^.

The change in ionic fraction between oxygen and metal ions is caused by migration of oxygen ions (vacancies) in the filament formed after ‘forming’ process within the resistance switching layer and can be described by the following equation of motion^2^

1 $$\frac{d\zeta (t)}{dt}=-\,{\mu }_{O}[V(t)-I(t){R}_{base}]/{L}^{2},$$

where ϛ(t) is the fraction of length of insulating volume to the total filament length L, R_base_ is the resistance of the base oxide layer, μ_O_ is the effective oxygen ion mobility within the filament that depends on electric field and state of the filament, and V(t) is the externally applied voltage^2^. I(t) is the current passing through the OxRAM which has ohmic relation at on-state whereas, at off-state, it basically follows the well-known Schottky barrier conduction model^12^. Then, I(t) at off-state (I_off_) for both polarities of voltage are represented as follows

2 $${I}_{off}(t)\approx \{\begin{array}{ll}{I}_{0}(t)\frac{e}{\eta kT}V(t), & {\rm{for}}\,V(t)\,\geqq \,{\rm{0}}\\ \frac{{I}_{0}(t)V(t)}{4{\varphi }_{B0}(t)+{I}_{0}(t)R(t)}, & {\rm{for}}\,V(t) < {\rm{0}}\end{array},$$

where I_0_(t)≡ I_00_exp[−eΦ_B_(t)/kT], I_00_ is the constant related to the filament cross-sectional area and temperature^1^, e is the unit charge, η is the ideality factor, and R(t) is the sum of R_base_ and resistance of the filament that depends on ϛ(t)^1,2^. Here, Φ_B_(t) is the Schottky barrier height that also depends on ϛ(t) and electric field that is modeled as^1,2^

3 $${\Phi }_{B}(t)\approx {\Phi }_{B0}(t)-\sqrt{\frac{q{E}_{m}}{4\pi \varepsilon }},{\Phi }_{B0}(t)={\Phi }_{B00}(1-\sqrt{1-\varsigma (t)}),$$

where E_m_ is the maximum electric field within the filament and Φ_B00_ is the maximum Schottky barrier height.

One of the notable points in Eq. (2) is that, if V(t) is positive with respect to the top electrode, as in the case of read operation, the resistance at off-state (mainly Poole-Frenkel conduction resistance) is nearly independent of the base layer resistance or the filament itself when the Schottky barrier is high enough than those resistances. Because the resistance at on-state simply equals to R_base_, then the on/off current ratio depends on R_base_ and the Schottky barrier height but independent with the resistance of filament.

**Figure 4:**
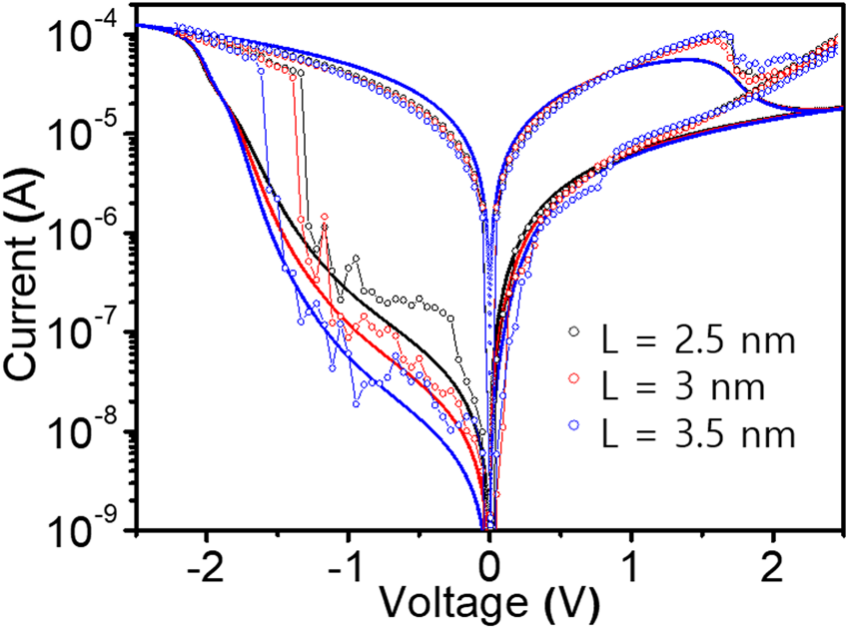
Results from the OxRAM switching model for different filament lengths (L = 2.5, 3, and 3.5 nm) with the experimental switching layer thickness conditions described in Fig. 1(b). Here, Φ_B00_ = 0.28 eV, the resistance of filament at off-state = 10^5^ Ω, the resistance of base layer = 2 × 10^4^ Ω, and μ_O_ for insulating volume and conducting volume are 3 × 10^−11^ and 3 × 10^−9^ m^2^s^−1^V^−1^ respectively.

Figure 4 shows the calculated I-V switching curves plotted from the switching model described above for different switching layer thicknesses together with the experimental results shown in Fig. 1(b). As can be seen in this figure, the model results have good agreements with the experiments both qualitatively and quantitatively for all the switching layer thicknesses. We can also notice that the on-state currents are almost invariant for different Ls and the asymmetry of off-state currents, which are one of the main features observed in OxRAMs^1,5^. The changes in currents at off-states can be figured out with the variation of oxide layer thickness and corresponding Schottky barrier (Φ_B00_) experimentally induced by changing number of ALD cycles as explained for Fig. 1(b).

Meanwhile, from Eq. (2), we can find out that ϛ(t) and on/off current ratio have the following relation

4 $$\sqrt{\varsigma (t)}=\sqrt{1-{(1-\frac{kT}{q{\Phi }_{B00}}{log}(on/off))}^{2}.}$$

And because the on/off current ratio at forward bias is equal to ${\rm{\eta }}\mathrm{kT}/{\rm{e}}{R}_{base}{I}_{0}$, log(on/off) can be expressed as

5 $$\mathrm{log}(on/off)\approx \frac{e}{kT}{\Phi }_{B00}(1-\sqrt{1-\varsigma (t)}),$$

And since ϛ(t) < 1, the standard deviation of log(on/off) can then be approximated and represented as

6 $$\frac{e{\Phi }_{B00}}{kT}\sigma (1-\sqrt{1-\varsigma (t)})\cong \frac{e{\Phi }_{B00}}{2kT}\sigma (\varsigma (t))=\frac{e{\Phi }_{B00}}{2kT}\frac{d}{L}\sigma (\frac{\varsigma (t)}{d}L)$$

where d is the lattice constant of insulating volume in the filament.

Because the validity of the approximation given in Eq. (6) is determined by whether we can ignore higher order terms of ϛ(t) in Taylor expansion and whether the difference between the real and the approximated values is acceptable that is automatically related to the question how sufficiently small ϛ(t) should be. From the fact that the range of log(on/off) we consider is between 0 and 3 (see Figs 2(b), 3 and 4), and with Eq. (4), we can find out that ϛ(t) varies from 0 to 0.5. In this range, it can be easily checked out that the real and the approximated values are proportional to each other with an error of less than about 15%.

It is well known that migration of ions in lattice of a solid is determined by the competition between the electric field with image charge force plus the statistical thermal energy and the migration barrier energy. In the meantime, the statistical distribution of the thermal energy of individual ions causes the migration to exhibit stochastic behavior and is the main cause of the resistance distribution^3^. In other words, in the case of switching the OxRAM from on-state to off-state (reset-operation), if oxygen ions in the filament averagely travel over a certain distance h, then it means they must go through averagely h/d (d is the lattice constant of the lattice) atomic barriers. During this process, some ions do not reach that distance, and some ions move further. This kind of diffusive transport makes oxygen ion migration in the filament of OxRAM essentially stochastic, resulting in distribution of resistance states to some extent.

Then in this situation, since the standard deviation of N probabilistic movements is $\sqrt{N}/2$^13^, and from Eq. (6), the standard deviation of log(on/off) simply can be expressed in terms of log(on/off) as

7 $$\sigma [\mathrm{log}(on/off)]=\frac{q{\Phi }_{B00}}{4kT}\sqrt{d/nL}{[1-{(1-\frac{kT}{q{\Phi }_{B00}}log(on/off))}^{2}]}^{1/2},$$

where n is the total number of migrating oxygen ions in the filament which can act as a proportionality constant to compensate for the fundamental error that Eq. (6) has. We roughly deduce the value of n as 10 from the filament area and the lattice constant.

Equation (7) again can be approximated by $\sqrt{qd{\Phi }_{B00}/2kTnL}\sqrt{log(on/off)}$/2 at the range of on/off ratio that we are interested in. From this equation, we can figure out that standard deviation of log(on/off) is proportional to square root of itself and to the maximum Schottky barrier energy divided by the temperature.

The principle that on-state exhibits a highly uniform resistance level as compared to off-states can be understood as that metals basically exhibit almost constant resistivities regardless of amount of impurities. In fact, if the amount of oxygen ions increases above a certain level, a metal-to-semiconductor (or insulator) transition occurs resulting in a significant increase in resistance, but that would be no longer an on-state but off-state.

**Figure 5:**
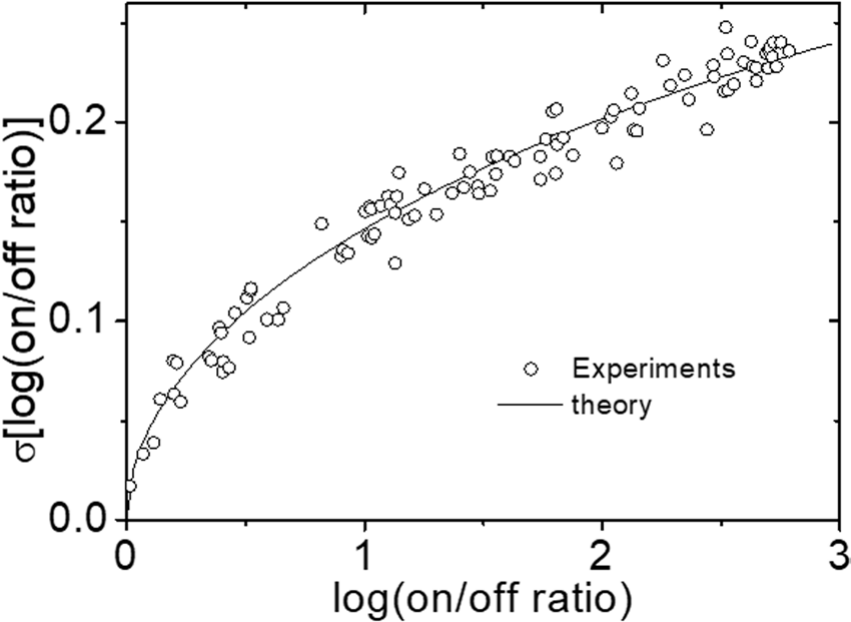
Theoretical predictions of standard deviation of log-scale on/off current ratio as a function of log-scale on/off current ratio from Eq. (6) with Φ_B00_ = 0.28 eV, d = 0.5 nm, L = 3 nm, and n = 10. To confirm the consistency, the experimental results with L = 3 nm presented in Fig. 3 are plotted together.

In Fig. 5 we plot calculated σ[log(on/off)] as a function of log(on/off) under the condition of L=3 nm and Φ_B00_ = 0.28 eV from Eq. (7) and compare with the experimental results shown in Fig. 3. Here, the value of Φ_B00_ is selected by matching model I-V curves as described in Fig. 4. As can be seen in the figure, the theory agrees quite well with the experimental results in the entire range of log(on/off) from near 0 to 3.

**Figure 6:**
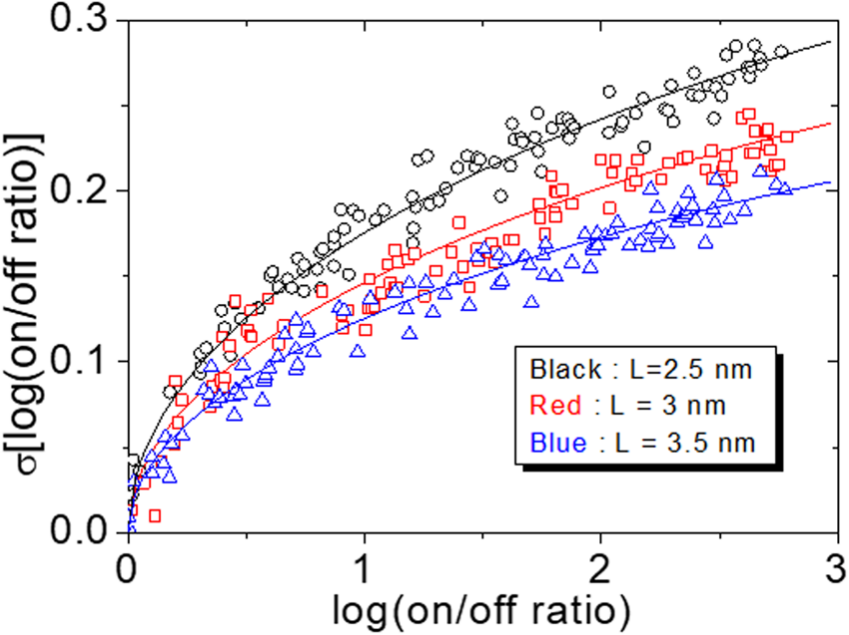
Experimental (dots) and corresponding theoretical calculation results (lines) of standard deviation of log-scale on/off current ratio as a function of log-scale on/off current ratio from Eq. (6) for different filament lengths (2.5 nm, 3 nm, 3.5 nm) which correspond to 40, 50, and 60 cycles of ALD respectively as explained in Fig. 1(b). The theoretical parameters are the same as Fig. 5 except L.

A further validity check of the theory is made by the experiments that compare between different thicknesses of resistance switching layer. In Fig. 6, we show the experimental and theoretical results of the resistance distribution tendencies as the switching layer thickness changes to 2.5, 3, and 3.5 nm while fixing all the other conditions as in Fig. 5. From the figure, we can see the remarkable agreement between the theory and the experiment. The dependence on the switching layer thickness surely comes from the fact that resistance state is determined by the fraction of insulating volume to L. Obviously, the longer the filament length, the smaller the impact of deviations of locations of oxygen ions. Another conclusion drawn from this result is that the higher the switching layer thickness, the higher the on/off ratio can be obtained with a smaller spread of resistance levels. However, in reality, if the layer thickness is larger than about 4 nm, stable resistance switching hardly can be achieved and futher study is needed to find out the cause of it.

At this point, let’s descriptively explain in more detail what the model means physically, which has been shown to describe the experiments very well. An irregular distribution of OxRAM resistance levels (or irregular distribution of oxygen partial volume in filament) can only be made by irregular responses of ionic migrations even under the same programming condition. This is relatively straightforward to understand, given that the ionic migration in a solid lattice essentially shows a stochastic, rather than deterministic, nature caused by phonon vibration under finite temperature. However, it is not straightforward to understand the fundamental principle of why deviation of resistances is proportional to on/off ratio of OxRAMs with very specific proportionality constants. We believe this can be understood phenomenologically in the following way.

In order to make a larger on/off ratio, the oxygen partial volume in the filament must be increased and thus a larger electric field must be applied to make oxygen vacancies travel longer distance in filament. Then, because probability of a series of stochastic events is multiplication of each probability, so as the distance of ionic migration increases, probabilistic uncertainty of single ionic migration builds up, resulting in a greater uncertainty that means larger deviation of resistance levels for OxRAMs. No matter how accurate this explanation may be, it is still surprising that, as we have shown above, the characteristics of resistance distribution of OxRAMs can be excellently explained both qualitatively and quantitatively by the basic principle of statistical physics when combined with the switching model of OxRAM.

This model is generally applicable if the OxRAM is multi-level implementation with a variation in resistance caused by ion migration within a filament of an oxide. Because majority of high-performance OxRAMs that have been implemented so far meet these conditions, we can safely say that this is the representative type of OxRAMs. Among such possible OxRAM types, we have studied with Ta_2_O_5_-based OxRAM because of its relatively stable and has the well-proven switching model.

To be more specific about universality, the statistical physics parts of this model have high generalities while, in order to apply the model to a particular type of OxRAMs, inevitably, specificity is reflected in the combined parts of the switching model of the OxRAM. Therefore, the model’s qualitative generality is maintained regardless of type of OxRAMs, and the value such as the proportional constant of the standard deviation of resistance levels can be affected by the material properties of the OxRAM. Specifically, values such as lattice constant d, oxygen ion density n, and Φ_B00_ in Eq. (7) are values that can be changed depending on the type of OxRAMs. However, since the values vary only within a certain range, it is strongly expected that there will not be a large change in the values even if the type of OxRAM is changed.

## Discussion

**Figure 7:**
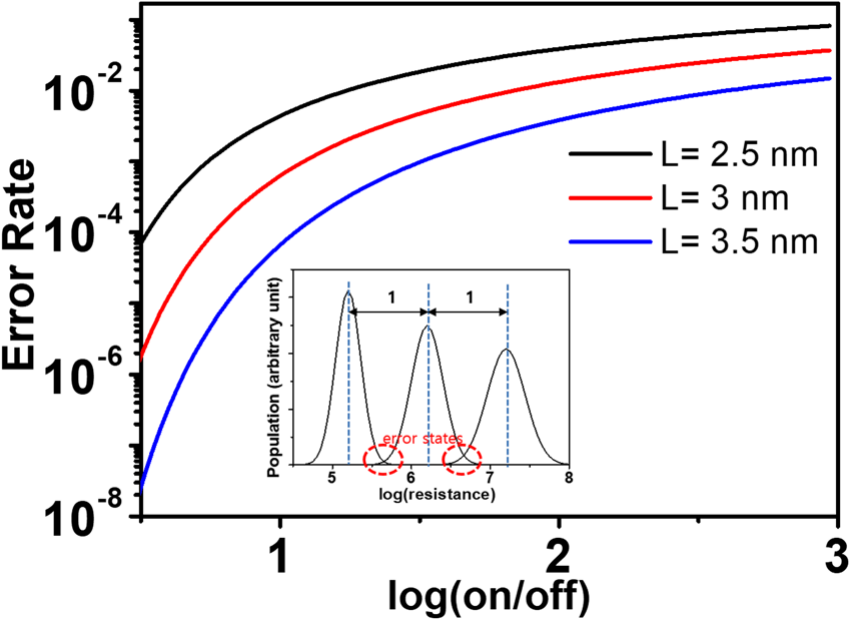
Error rates for a multiple resistance-state programmed state with two neighboring states as a function of log(on/off) for several switching layer thicknesses. The theoretical parameters are the same in Fig. 5.

It would be meaningful to discuss the effect of the resistance distribution characteristics of the unit OxRAM cell discussed so far on programming error rate of the device. As we have seen so far, when programming OxRAM at a multiple resistance-level mode, distributions of resistance levels from the targeted resistances are proportional to on/off ratio, which is certainly bad news in that a higher on/off current ratio is preferred. But how does this spread of resistances affect programming error? Let us calculate the read rate due to the overlaps of the resistance levels. Assuming that the distributions of resistance level are normal distributions, a certain state of resistance inevitably overlaps with the adjacent resistance states. The inset of Fig. 7 shows the illustration of this situation for the interval between adjacent resistance levels equals to 1 at log-scale. As marked with dotted red lines in the inset, the overlapped states are error states erroneously read as either of adjacent states. If we assume that overlapping occurs at approximately the middle of two resistance levels, the resistance level that is read incorrectly can be obtained by the following equation with an error function as follows

8 $$Error\,rate\approx 1-erf(\frac{1}{2\sqrt{2}\sigma }),$$

and the error rates for different switching layer thicknesses (L) calculated by this equation are plotted in Fig. 7. As can be seen in the figure, the error rate is a function that varies sensitively to both on/off ratio and switching layer thickness. Looking at the case of L = 3.5 nm, the standard deviation of the resistance level increases only about 0.04 while the log (on/off) increases from 1 to 2, but the programming error rate increases almost 100 times. And in the case of log (on/off) = 2, we can see that the error rate increases by 10 times when L varies from 3.5 to 3 and 2.5 nm. Unlike the dependence of resistance distributions, which showed relatively modest changes, the highly sensitive dependence of the programming error rate on both on/off ratio and L reveals why resistance level distribution is a significant problem in actual memory devices.

Among the memory devices currently being manufactured or referred to as candidates for next-generation memory devices, only resistive memories including OxRAM has a switching mechanism mainly based on ionic transport. More specifically, some kind of memory devices such as NAND flash, DRAM, and magnetic RAM (MRAM) are based on electronic phenomena with no (or only one) barrier, while others involve atomic migration, but are basically limited to local atomic reconfiguration. This unique feature in operating principle can create the advantages of OxRAMs, but at the same time it probably makes the poor distribution characteristics of resistance levels which can possibly impose limitation on its application of high-capacity memory device.

In summary, we have presented a theoretical model that successfully explains the experimentally measured characteristics of resistance level distributions in OxRAM. This model might provide useful insight to more clearly understand the potential of OxRAM to be a powerful high-capacity memory device. Besides, it also might be an important basis for finding ways to mitigate the problem that should be done in the future

## Methods

### Preparation of Ta_2_O_5−x_/TaO_2−x_ devices

The Pt bottom electrode was deposited using DC magnetron sputtering with a metal target and a Ta target in varying partial oxygen atmospheres at 400◦ was used to deposit the base layer with about 40 nm thickness. The insulating Ta_2_O_5−x_ layer was formed by placing our samples next in a plasma oxidation chamber used for atomic layer deposition. We flowed 300 s.c.c.m. of oxygen into the chamber using radiofrequency plasma. The 30-nm-thick Pt top electrodes were deposited using the same process as the bottom electrodes. Device sizes of 0.5 × 0.5 μm^2^ were fabricated by a conventional photolithography and a liftoff process.

### Electric measurements

The slowly sweeping electrical measurements were carried out by an Agilent 4156 C semiconductor analyser in voltage-sweep mode. Pulse measurements for repeated cycles were conducted by an Agilent 81110 A pulse generator and Tektronix oscilloscope (6 GHz).
